# Editorial

**Published:** 1872-04

**Authors:** 


### Editorial.

***Hereafter—***

The JOURNAL will be provided with an ample proportion of Editorial and Original Communications. Owing to causes over which we had no control, the present Number goes out with little save selected matter. The monthly issue is within a few days of date, and from and after this month it will be promptly on time.

Especial pleasure would be given both editors and readers, if many new correspondents would volunteer from among our numerous, intelligent and experienced subscribers.

